# A Rare Case of Transient Mesenteric Ischemia After Atrial Fibrillation With Rapid Ventricular Response Rate

**DOI:** 10.7759/cureus.58210

**Published:** 2024-04-13

**Authors:** Diva Maraj, Bipneet Singh, Rachael Fuller, Merritt Bern

**Affiliations:** 1 Internal Medicine, Henry Ford Health System, Jackson, USA; 2 Internal Medicine, Henry Ford Jackson Hospital, Jackson, USA; 3 Gastroenterology, Henry Ford Jackson Hospital, Jackson, USA; 4 Gastroenterology and Hepatology, Henry Ford Health System, Jackson, USA

**Keywords:** superior mesenteric artery occlusion, superior mesenteric artery, emboli, anti coagulation, mesenteric ischemia, atrial fib

## Abstract

Mesenteric ischemia is an urgent event and requires prompt recognition and treatment, in order to reduce the risk of mortality. It results from the sudden onset of small intestinal hypoperfusion, from a reduction or cessation of arterial perfusion, which can occur from an embolic obstruction at the superior mesenteric artery. We present a case of transient mesenteric ischemia from an episode of atrial fibrillation with a rapid ventricular response rate. Despite being on chronic anticoagulation therapy, the patient developed transient mesenteric ischemia from an embolic clot. The patient's heart rate was controlled and no surgical intervention was required, a rare finding; however, it is very important to recognize and treat promptly.

## Introduction

Mesenteric ischemia can occur from an embolic obstruction, which is the most common cause, and usually occurs at the superior mesenteric artery (SMA) or from mesenteric arterial thrombosis [1-2]. Acute mesenteric ischemia must be confirmed, with computed tomography (CT) of the abdomen, as these patients require urgent surgical intervention [1]. There is a large component of patients with embolic disease, such as atrial fibrillation who present with acute mesenteric ischemia. Atrial fibrillation is closely related to atherosclerosis and can compound ischemia through the vascular system by accelerating atherosclerosis, resulting in thrombus formation, with resultant embolization [3-4]. In these patients, especially those presenting a rapid ventricular response rate, it is important for rate control as well as anticoagulation. In our patient, after controlling for rate, his transient mesenteric ischemia resolved and he was resumed on anticoagulation. This case presents a rare finding of transient mesenteric ischemia and different approaches to management, including gastroenterology and vascular surgery consultation.

## Case presentation

**Figure 1:**
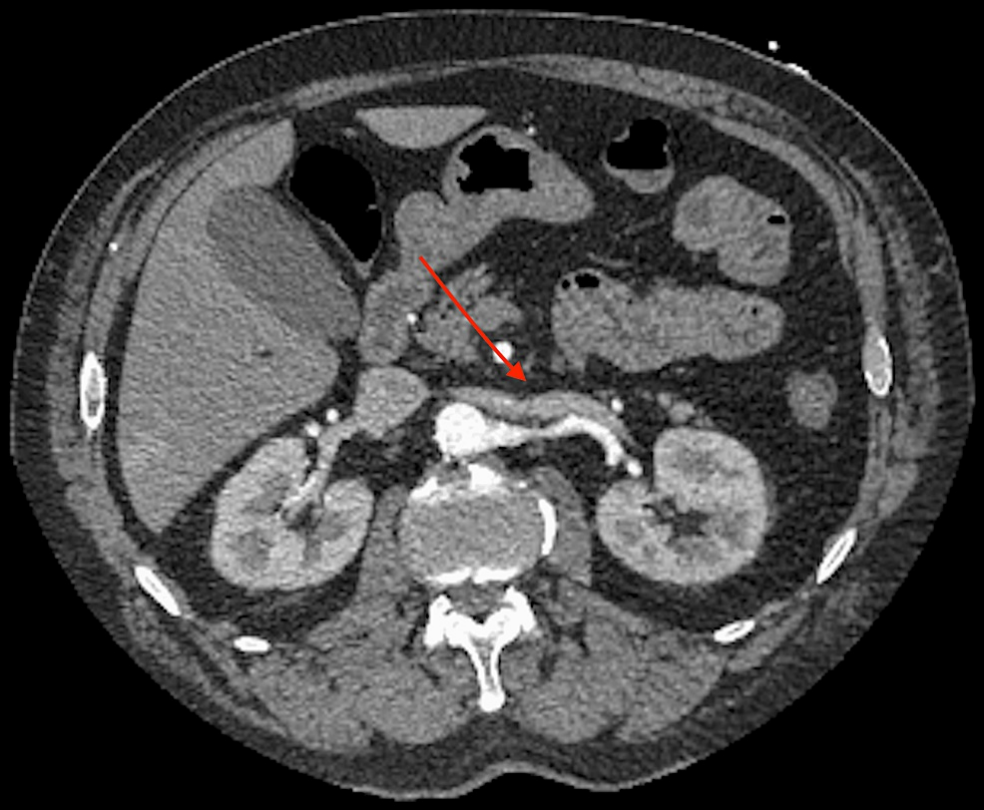
CT scan showing superior mesenteric artery stenosis as indicated by red arrow.

A 65-year-old male presented to the hospital complaining of abdominal pain. The patient stated that he had diffuse abdominal pain, mainly localized at the periumbilical region. He stated that the pain started the morning prior to presentation, with nausea, vomiting, and palpitations. On physical examination, the patient had abdominal pain out of proportion to examination, with localized tenderness to the periumbilical region. He also had an irregular heart rate with tachycardia, no murmurs, gallops, or rubs were heard. The patient had a history of paroxysmal atrial fibrillation on apixaban, coronary artery disease status post-percutaneous intervention to the left anterior descending artery, only on aspirin in addition to apixaban, chronic obstructive coronary disease, and lung cancer, status post resection of the left upper lobe. Differentials at this time included atrial fibrillation with rapid ventricular response rate, mesenteric ischemia, gastritis, peptic ulcer disease, diverticulosis, acute coronary syndrome, pulmonary embolism, hyperthyroidism, or hypothyroidism. When the patient presented, he was found to be in atrial fibrillation with a rapid ventricular response rate in the 160’s, as seen on the electrocardiogram. On labs, he was found to have an elevated lactate of 2.8. An abdominal CT was also obtained, which showed SMA stenosis of 60% (Figure 1). The patient was started on a diltiazem drip, and his anticoagulation, apixaban was resumed, and the patient’s heart rate improved. The patient was also started on intravenous fluids, which improved the patient’s lactate. The patient was also seen by cardiology, who increased his home dose of metoprolol succinate and agreed to maintain anticoagulation. The patient also underwent an esophagogastroduodenoscopy (EGD), which showed gastritis and duodenitis, which was biopsied to look for any changes of ischemia, which were negative. The patient underwent a repeat CT angiography of the abdomen and pelvis, which showed 75% stenosis of the SMA. Abdominal visceral imaging also showed greater than 70% stenosis in the proximal SMA. The patient was seen by vascular surgery who recommended no intervention and follow-up with repeat mesenteric duplex in six months. The patient continued to improve and was discharged home in stable condition, with repeat appointments with cardiology and vascular surgery scheduled. It is likely that the patient experienced transient mesenteric ischemia from his episode of atrial fibrillation with rapid ventricular response rate, leading to a possible embolization and stenosis of the mesenteric artery, which resolved once his heart rate improved. It was stressed that the patient should continue heart rate management medications, including his apixaban.

## Discussion

Mesenteric ischemia results from the sudden onset of small intestinal hypoperfusion, from a reduction or cessation of arterial perfusion. This can occur from an embolic obstruction, which is the most common cause, and usually occurs at the SMA or from mesenteric arterial thrombosis. Embolism to the mesenteric arteries can occur when a thrombus gets dislodged, likely from the left atrium, left ventricle, cardiac valves, or proximal aorta. Acute mesenteric ischemia is a life-threatening emergency with a very high mortality rate between 50 and 70% [1-2]. Patients usually present with periumbilical abdominal pain, out of proportion to physical exam findings and can have laboratory abnormalities including lactic acidosis or an anion gap metabolic acidosis [1-2]. Early diagnosis and intervention are crucial to reduce mortality. A CT angiogram is the gold standard used to diagnose mesenteric ischemia [1,5-7]. The current guidelines for the management of mesenteric ischemia include surgical endovascular repair [5].

Patients with atrial fibrillation can develop thromboembolic acute mesenteric ischemia, even when fully anticoagulated. Atrial fibrillation is one of the known common arrhythmias associated with mesenteric ischemia, and a high rate of embolization is seen in these patients [2]. During this patient’s admission, he was also in an episode of atrial fibrillation with a rapid ventricular response rate, and the decision was made to continue anticoagulation and optimize rate control medication. In these patients, guidelines recommend using either intravenous beta-blockers or calcium channel blockers to rapidly control the heart rate [6]. It was noted that once the patient’s rapid ventricular response rate was controlled, his abdominal pain greatly improved. This patient was prone to transient mesenteric ischemia because of his non-critical 60% SMA stenosis, likely due to a small embolus from his atrial fibrillation with a rapid ventricular rate. Our patient’s mesenteric ischemia was transient as it resolved with thrombus passage or spontaneous dissolution; however, if this patient had 95% stenosis, he would have had a higher risk of morbidity and mortality.

## Conclusions

Management of patients with transient mesenteric ischemia from atrial fibrillation with rapid ventricular response rate includes resuming anticoagulation and ensuring that heart rate control has been achieved, to limit the extent of embolization, which was seen to be successful in our case. If prompt clinical presentation, such as abdominal pain or laboratory markers, including persistent elevated lactic acidosis are present, then it’s imperative that the patient receives urgent endovascular repair. This case report highlighted the importance of recognizing a transient episode of acute mesenteric ischemia in patients with atrial fibrillation presenting with rapid ventricular response rate, and the importance of prompt diagnosis and imaging. As this is one of the only case studies documenting this clinical presentation, further research should be conducted for recommended treatment and management.
